# Immunohistochemical analysis of cancer-associated fibroblasts and podoplanin in head and neck cancer

**DOI:** 10.4317/medoral.23335

**Published:** 2020-01-22

**Authors:** Víctor Ramos-Vega, Bernardo Venegas Rojas, Wendy Donoso Torres

**Affiliations:** 1 DDS, MSc. Department of Basic Biomedical Sciences, Faculty of Health Sciences, University of Talca, Talca, Chile; 2 DDS, MSc, PhD. Department of Stomatology, Faculty of Health Sciences, University of Talca, Talca, Chile; 3 MSc, PhD. Department of Stomatology, Faculty of Health Sciences, University of Talca, Talca, Chile

## Abstract

**Background:**

To immunohistochemically evaluate the association between the presence of cancer-associated fibroblasts (CAFs) and the tumour expression of podoplanin (PDPN) in head and neck squamous cell carcinoma (HNSCC) and their association with clinicopathological variables.

**Material and Methods:**

A tissue microarray (TMA) with biopsy sections from patients diagnosed with HNSCC was stained with antibodies against the CAFs marker, α-smooth muscle actin (α-SMA), and PDPN. We subsequently evaluated their expression to determine the association between them and with clinicopathological variables including age, primary tumour site, TNM stage, and tumour differentiation grade.

**Results:**

Positive reaction to α-SMA was observed in the tumour stroma, revealing spindle-shaped cells compatible with CAFs, which showed a high expression in 62% of cases and a significant association with laryngeal carcinomas, advanced clinical stages, and lower tumour differentiation (*P* ≤ 0.05). PDPN staining on tumour cells showed low expression in 72% of cases, and it was not associated with any clinicopathological variable or with the presence of CAFs.

**Conclusions:**

The presence of CAFs in the tumour stroma is related to an aggressive phenotype and could increase as the disease progresses, although based on our findings, it would have no relationship, at least directly, with the expression of PDPN.

** Key words:**Cancer-associated fibroblasts, myofibroblasts, head and neck neoplasms, podoplanin, immunohistochemistry.

## Introduction

Head and neck cancer (HNC), which is the sixth most common form of cancer in the world, comprises a heterogeneous group of malignant neoplasms, with more than 90% of cases corresponding to squamous cell carcinoma (SCC) originating in the epithelium of the mucosal lining of the oral cavity, oropharynx, larynx, hypopharynx, and nasopharynx (1,2). Despite therapeutic advances, HNC has a five-year survival rate of approximately 50%, mainly due to late diagnosis (3).

Currently, the aim is to identify biomarkers that allow us to understand the molecular mechanisms associated with tumour development to investigate therapeutic targets that limit the progression of the disease and improve the patient’s prognosis (4). One of the most studied biomarkers is podoplanin (PDPN), a transmembrane glycoprotein that participates in multiple physiological processes, such as the formation of lymphatic vessels, lung development, nervous system development, and immune system activity (5). In pathology, several studies have shown that the expression of PDPN in premalignant lesions of the oral cavity and larynx is associated with a higher degree of dysplasia, higher risk of malignant transformation to SCC, and low survival, so it would play a key role in carcinogenesis (6,7). On the other hand, it has been demonstrated that tumour expression of PDPN in head and neck SCC (HNSCC) is associated with advanced forms of the disease, metastases in regional lymph nodes, and low survival (8). Specifically, the activation of PDPN in tumour cells would stimulate invadopodia formation and degradation of the extracellular matrix, processes that promote epithelial-mesenchymal transition (EMT) and tumour metastasis (9).

The study of cancer over the last few decades has focused on the participation of the tumour stroma in its development and progression, since it presents a wide cellular and molecular heterogeneity derived from an active interaction between the neoplasm and the host tissues. It has consequently been defined as the tumour microenvironment (10). In this context, several tumours show a marked presence of cancer-associated fibroblasts (CAFs), an activated form of the fibroblasts present in the tumour stroma that are characterized by the expression of α-smooth muscle actin protein (α-SMA) and that would stimulate the invasive behaviour of the carcinoma through reciprocal interactions (11). Some studies have shown that the presence of CAFs is associated with local recurrence of the disease and low survival (11,12,13), as they would have the ability to induce an aggressive tumour phenotype through the expression of growth factors, cytokines, chemokines, and proteolytic enzymes such as metalloproteinases. However, all the molecular mechanisms regulated by CAFs in the context of HNSCC have not yet been established (11).

Evidence exists that the tumour cells of oral cavity SCC have the capacity to induce the differentiation of CAFs and that these in turn would induce the expression of PDPN in tumour cells through the secretion of TGF-β, favouring EMT and invasiveness of the carcinoma (14). This indicates that the relationship between CAFs and the tumour expression of PDPN could play a significant role in the progression of HNC. In this context, the aim of this study is to evaluate, through immunohistochemistry, the presence of CAFs and their association with tumour expression of PDPN in biopsies obtained from patients diagnosed with HNC, and to evaluate the association of both with clinicopathological variables.

## Material and Methods

- Sample

We conducted a retrospective study over a sample of formalin-paraffin fixed tissue sections obtained from biopsies of patients diagnosed with HNSCC, purchased in a tissue microarray format (TMA; Biomax, Derwood, USA). A TMA comprises 60 tissue sections, of 5 µm thickness, from primary tumours collected from 30 HNSCC patients (two specimens per case) and 21 tissue sections from normal mucosa, serving as control. Of the 30 primary tumours included on the TMA, 16 came from the larynx, 9 came from the oral cavity, and five came from the nasosinusal mucosa. One of the oral cavity primary tumour specimens did not meet the histopathological criteria for the diagnosis of squamous cell carcinoma, and it was excluded from the study.

Clinical and histopathological data from the 29 HNSCC patients are presented in Table 1 and summarized in Table 2, including age, gender, TNM clinical staging, lymph node compromise, and tumour differentiation grade. The clinical stage was stablished according to the TNM staging system outlined by the American Joint Committee on Cancer Staging Manual, 7th edition. Tumour differentiation was performed according to the Broder’s classification system.

Patients ranged from 34 to 81 years (mean 57 years) in age, and 82.7% of them were male. Of the 29 primary tumours analysed, 12 were early stage I/II and 17 advanced stage III/IV; 4 had associated cervical lymph node metastases and 25 were non-metastatic; and 12 were classified as differentiated tumours (G1) and 17 as undifferentiated tumours (G2 or G3).

This research was approved by the ethical committee of the University of Talca with the approval code 2017-01-BV-VR on april 2017. Nevertheless, the human tissue samples were acquired in a commercial format by biomax (Biomax, Derwood, USA), who declare that all tissue is collected under the highest ethical standards with the donor being informed completely and with their consent. They ensure that follow standard medical care and protect the donors' privacy. They also declare that all human tissues are collected under HIPPA approved protocols, have been tested negative for HIV and Hepatitis B and approved for commercial product development.

**Table 1:**
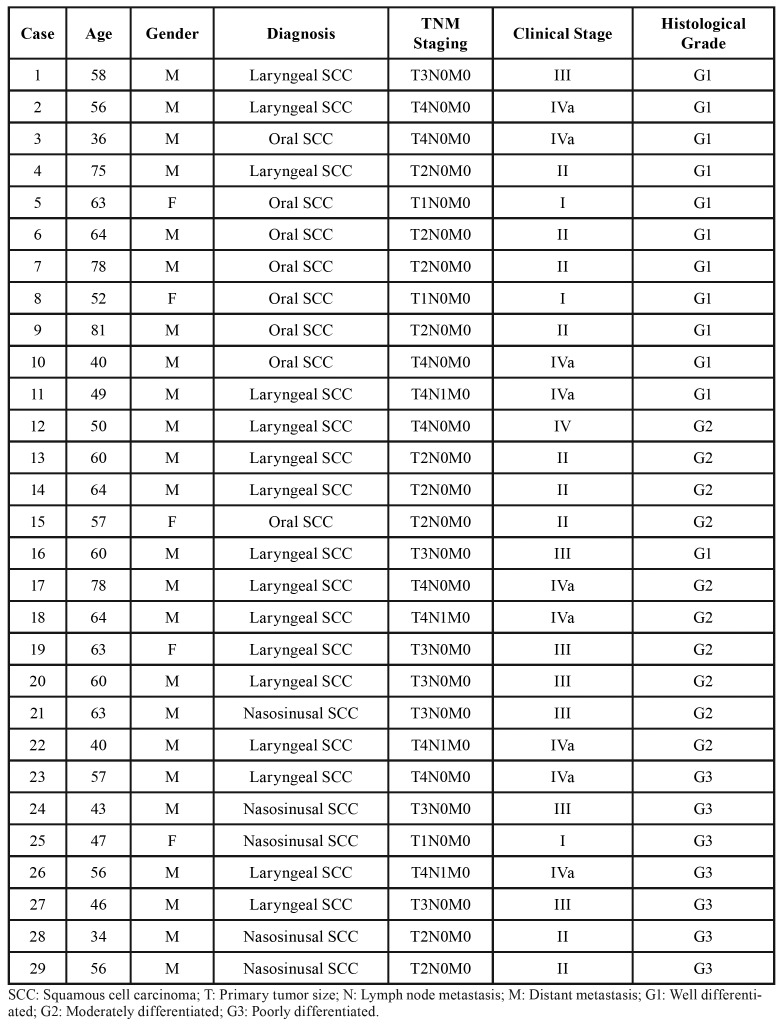
Clinical and histopathological variables of HNSCC patients.

**Table 2:**
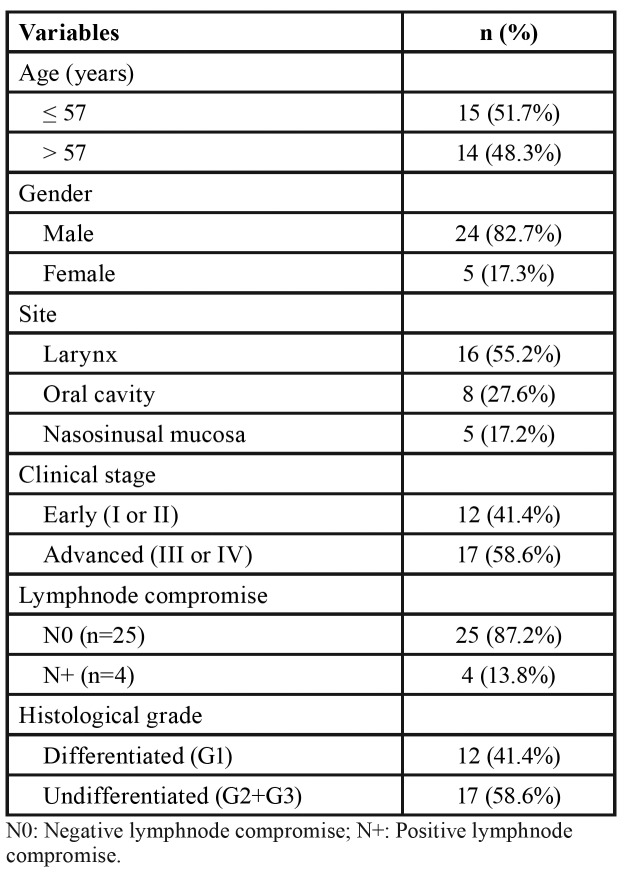
Summary of clinocopathological variables of HNSCC patients.

- Immunohistochemistry

The TMA were dewaxed in xylene and rehydrated in graded alcohols. After a rinse in tap water, sections were immersed in a 3% hydrogen peroxide solution for 10 min. Slides were placed on a steamer at 95 °C for 30 min in Tris-EDTA buffer solution (pH 9.0) for antigen retrieval and th*en bloc*ked with 1% bovine serum albumen for 10 min. Next, sections were incubated with anti-α-SMA (Agilent Technologies Dako, California, USA; 1:100 dilution) and anti-PDPN (Agilent Technologies Dako, California, USA; 1:50 dilution) primary mouse antibodies for 1 h at 37 °C and washed in phosphate-buffered saline (PBS) three times for 2 min. Sections were incubated with secondary biotinylated antibody at room temperature for 30 min in accordance with the manufacturer’s instructions (BA2000, Vector Laboratories, California, Estados Unidos). All slides were then exposed to avidin-biotin complex and diaminobenzidine tetrahydrochloride (Sigma, St. Louis, MO) and counterstained with Harris haematoxylin. Internal positive controls were used for each antibody: smooth muscle cells of blood vessels for α-SMA and lymphatic endothelial cells of lymphatic vessel for PDPN. For negative control, the primary antibody was substituted with phosphate-buffered saline.

- Immunohistochemical analysis

The IHC-stained TMA sections were observed under a light microscope (Leica DM500, Leica Microsystems, Wetzlar, Alemania) and the images for the analysis were obtained through an integrated digital camera (Leica IC500, Leica Microsystems, Wetzlar, Alemania). Evaluation of immunostaining for both antigens was performed by two observers independently (V.R-V. and B.V.) who were blinded to the clinical information pertaining to the subjects. Cases with interobserver discrepancies were minimal and reassessed together until a consensus was reached.

Cancer-associated fibroblasts were defined as spindle-shaped fibroblasts on tumour stroma expressing α-SMA as described previously (15). The presence of CAFs was classified into four grades, based on a previously described classification system (16), as negative (score 0), no CAFs; scanty (score 1), a small number of scattered CAFs; focal (score 2), concentrated CAFs with an irregular and non-continuous distribution; and abundant (score 3), concentrated CAFs with an extensive and continuous focus surrounding the tumour. Each specimen was scored at the highest grade throughout the entire invasive stroma. First, grades 2 and 3 were distinguished from other grades at 40x and 100x magnification. Second, if no evident concentrated foci of CAFs were observed at low magnification, grades 0 and 1 were assessed at 400x magnification. For statistical analysis, grades 3 and 2 were categorized as high expression of α-SMA, while grades 0 and 1 were categorized as low expression of α-SMA.

For scoring the immunoreactivity of PDPN, five representative fields at the tumour invasive front of each case were selected under 400x magnification and evaluated on ImageJ 1.47q (NIH®) software, following a previously described method (17). Membrane immunoreactivity on the tumour cells of the carcinoma was considered to indicate PDPN expression. Quantity scores from 0 to 5 were given if 0%, 1% to 10%, 11% to 30%, 31% to 50%, 51% to 80%, and 81% to 100% of the tumour cells were positive on each field, respectively. The staining intensity of the whole sample was rated on a scale of 0 to 3, with 0 as negative, 1 as weak intensity, 2 as moderate intensity, and 3 as strong intensity. The data were then converted to immunoreactive scores (IRS) by multiplying the average quantity scores of the five selected fields and the staining intensity scores of the sample. IRS values can range from 0 to 15. For statistical analysis, IRS values greater than 7 were categorized as high expression of PDPN, while IRS values equal to or less than 7 were categorized as low expression of PDPN.

- Statistical analysis

The Spearman correlation coefficient was used to test the association between the staining scores of α-SMA and PDPN. The association between the expression degree of each antigen and between each antigen and the clinicopathological variables was made by the chi-square test. All differences were considered significant if *P* ≤ 0.05. The statistical tests were performed using SPSS software, version 23.0.0.0 (IBM, North Castle, New York, USA).

## Results

- Immunohistochemical findings

**Figure 1:**
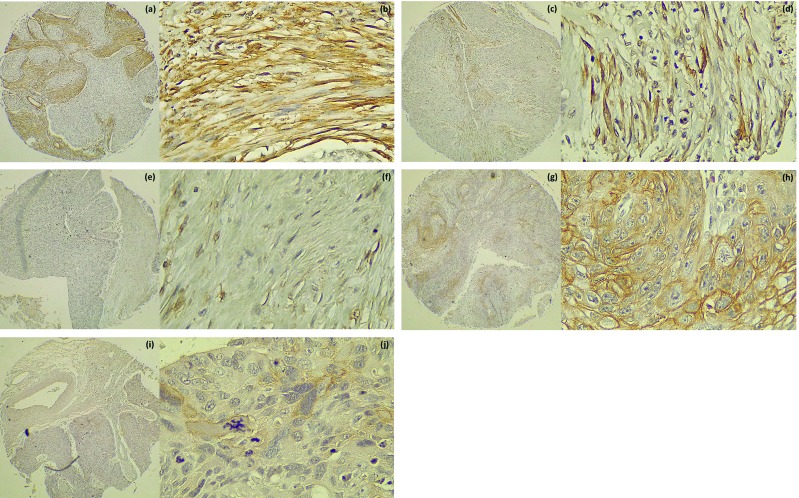
Immunohistochemical staining patterns for α-SMA at 40x and 400x magnifications: (a,b) abundant; (c,d) focal; (e,f) scanty. Immunohistochemical staining for PDPN at 40x and 400x magnifications: (g,h) high expression; (i,j) low expression.

All cases showed a positive reaction for α-SMA staining in stromal cells, which showed a cytoplasmic staining pattern that allowed us to observe spindle-like cells, whose morphology is compatible with CAFs. As summarized in Table 3, 62% of the cases showed a high degree of α-SMA expression, while 38% showed a low degree of α-SMA expression in the tumour stroma. Fig. 1 shows representative images of each of the IHC staining patterns for α-SMA; none of the samples had a score of 0.

**Table 3:**
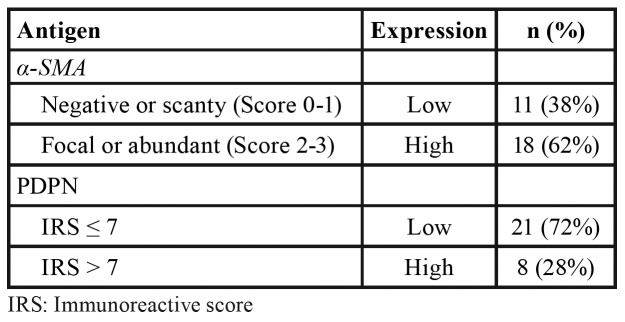
Immunohistochemical evaluation of α-SMA and PDPN.

In most cases, we observed a low degree of PDPN expression (72%, Table 3), with three samples showing a negative reaction (IRS = 0).The staining was observed at the tumour invasive front, where epithelial tumour cells showed a membrane staining pattern at the basal and suprabasal layers of the epithelium. Some cases showed a positive reaction on cells in the stroma, which there were not considered for the purposes of this study. Fig. 1 shows representative images of high- and low-PDPN expression degrees.

- Relationship between α-SMA, PDPN, and clinicopathological variables

**Figure 2:**
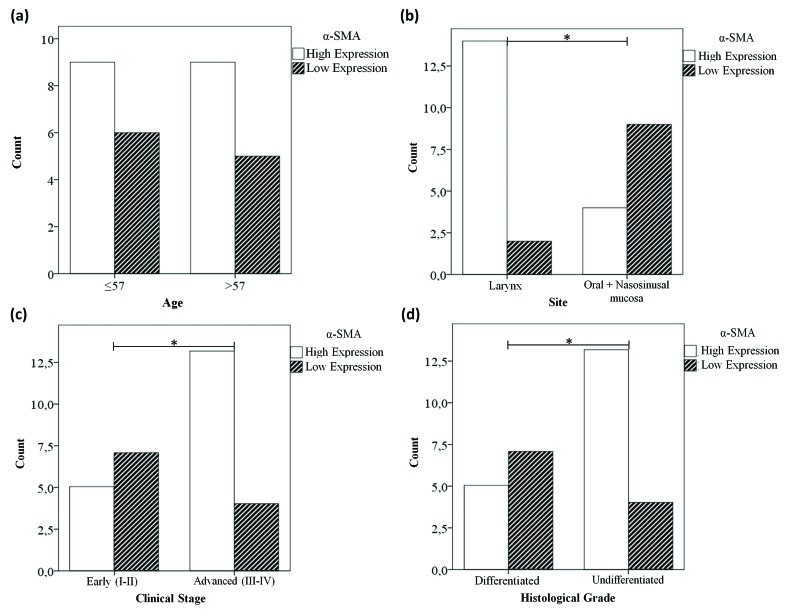
Expression degrees for α-SMA associated with clinicopathological variables (* *p* ≤ 0.05). While there is no association between α-SMA and age (a), a significant association is observed with laryngeal carcinomas (b), advanced clinical stages (c) and lower tumor differentiation (d).

Table 4 shows the contingency Tables used to establish the association between the degrees of expression of each of the antigens evaluated and the clinicopathological variables. A high expression of α-SMA is significantly associated with advanced clinical stages of the disease and a lower degree of tumour differentiation (x2, *p* ≤ 0.05); notably, 76% of patients in stages III–IV or who present undifferentiated tumours have a high α-SMA expression degree. In addition, laryngeal SCC showed a higher α-SMA expression degree compared with oral cavity and nasosinusal SCC (x2, *p* ≤ 0.05). These findings are graphed on Fig. 2. We did not find an association between PDPN expression degree with α-SMA expression or with any of the clinicopathological variables evaluated, so the PDPN data were not graphed.

**Table 4:**
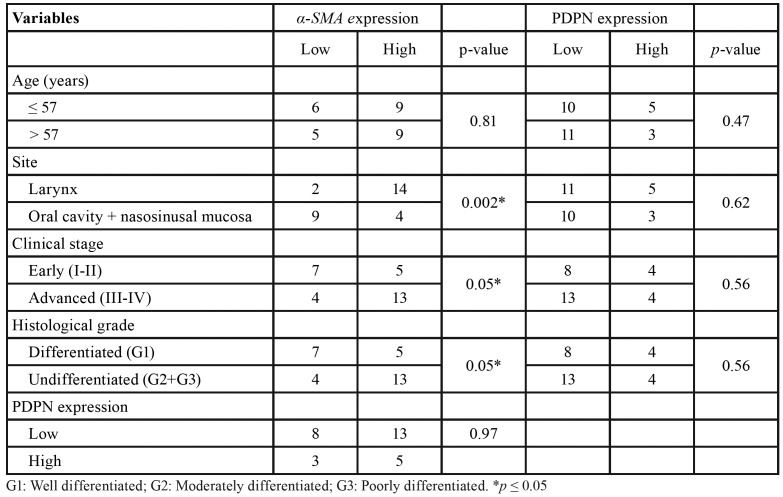
Relationship between α-SMA, PDPN and clinocopathological variables.

**Figure 3:**
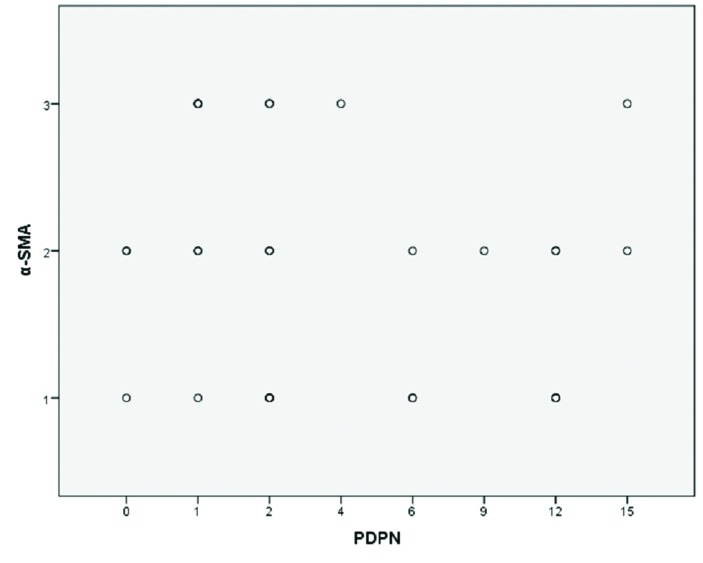
Linear scatter plot for the immunostaining scores of α-SMA and PDPN that show no correlation between them (Spearman, *p* = 0.49).

Fig. 3 shows the linear scatter plot for the immunostaining scores of α-SMA and PDPN, where it can be seen that there is no trend showing a linear correlation between the immunostaining values for each antigen. The distribution of the values for both variables does not conform to normal distribution (Shapiro-Wilk), so a Spearman correlation test was applied to confirm that there is no correlation between the immunostaining values of α-SMA and PDPN (*p* = 0.49).

## Discussion

Currently, the management and treatment of HNSCC depends mainly on clinical aspects, where the TNM classification system is highlighted and complemented with histopathological information specific to the tumour and nodal involvement. Despite advances in the study of pathogenesis and treatment, the survival of patients diagnosed with this type of cancer has not improved significantly in recent years (1,18). Although clinical parameters facilitate therapeutic decisions and the estimation of prognosis, knowing the biological and molecular mechanisms that accompany the development of the neoplasm is relevant to identify new therapeutic targets to develop complementary therapies that improve the course of the disease.

In the tumour microenvironment of the HNSCC there is a marked presence of CAFs, which participate in processes that promote tumour progression, such as lymphangiogenesis, angiogenesis, modulation of the immune system, and promote the proliferation and migration of tumour cells (15,19-21), clinically related to a low survival and poor prognosis (22). The presence of CAFs can be assessed by the identification of several antigens, although the α-SMA protein has been shown to be one of the most commonly used markers to distinguish this cell type from normal fibroblasts and other stromal cells (21).

In our sample, we evaluated the immunohistochemical expression of α-SMA in biopsies of patients diagnosed with SCCHN, which allowed us to identify CAFs in the tumour stroma and evaluate its distribution. In all cases there was positive expression of α-SMA, with a predominance of focal and abundant patterns (62%), associated with advanced clinical stages (III, IV, IVA) and a lower degree of tumour differentiation. Previous studies have shown the influence of CAFs on the development of SCCHN, associating the expression of α-SMA with aggressive anaplastic tumours, advanced clinical stages, regional lymph node involvement, and recurrence of the disease (20,23). Moreover, CAFs has been outlined as a possible prognostic factor, considering that the inhibition of its activity has been shown to limit the progression of SCC *in vitro* and *in vivo* (24,25). Additionally, in our sample we observed a significant association between the expression of α-SMA and the primary tumour site, where cases of laryngeal SCC showed a greater presence of CAFs compared with oral SCC and nasosinusal SCC. There are no studies comparing the expression of α-SMA between different primary tumour sites in HNSCC; however, this difference can be explained due to the association observed between the presence of CAFs and the clinical stage, considering that 80% of laryngeal SCC cases were in advanced clinical stages, while only 30% of cases of oral and nasosinusal SCC were in these stages.

Among the possible mechanisms involved in the interaction between the tumour microenvironment and carcinoma, it has been established that CAFs would induce the expression of proteins that favour the EMT of tumour cells such as PDPN, which has been outlined in recent years as a possible biomarker of tumour progression (4). PDPN is expressed mainly in the tumour invasion front (17,26), and its biological role would be related to the collective migration of tumour cells, which implies the loss of its adherent junctions to neighbouring epithelial cells and its subsequent invasion towards the underlying connective tissue, promoting metastasis (8).

Most of the studies that evaluate the immunohistochemical expression of PDPN and its relationship with clinicopathological variables have been carried out on oral SCC, where a greater expression of PDPN is associated with low survival and worse prognosis (7,17). In addition, PDPN showed a greater expression in oral SCC than in premalignant lesions, which in turn present a higher expression in cases with severe dysplasia (6,7), so PDPN is emerging as a possible biomarker of carcinogenesis and neoplastic development in early stages (6,26). In other forms of HNSCC this association is variable, whereas a similar relationship has been observed between tumour expression of PDPN with low survival and infiltrating phenotype in laryngeal and oesophageal SCC (27-30). In other sites, such as the oropharynx, this association could not be demonstrated (31). These differences can be explained because there are different risk factors and site-specific molecular mechanisms for each carcinoma. Although in our investigation we could not demonstrate the association between PDPN with clinicopathological parameters, we observed a positive expression in most cases, which accounts for its possible role in carcinogenic mechanisms, so it would be interesting to continue inquiring about its biological role.

In this research we could not demonstrate any significant association between the presence of CAFs and the tumour expression of PDPN. However, recent studies have shown that oral SCC tumour cells induce the differentiation of CAFs through the production and release of TGF-β and, in turn, CAFs through the secretion of the same factor, which induces both the differentiation of new CAFs and the expression of PDPN in tumour cells (14). In vitro and *in vivo* studies have shown that CAFs induce the expression of PDPN in oral SCC cell lines whose activation is associated with the formation of invadopodia and degradation of the extracellular matrix by activating the insulin-like growth factor-II mRNA binding protein-3 (IMP-3) pathway, causing the tumour cells to acquire an invasive phenotype with migratory capacity, favouring EMT and metastasis (9), which indicates a potential therapeutic target that is of interest in developing novel experimental and histopathological studies that seek to clarify this relationship in different forms of HNSCC.

The limitations of the study were the small sample, the use of TMA acquired in commercial format where there is no certainty that the sections obtained are representative of the original biopsy and the lack of functional tests confirming that CAFs induce tumor expression of PDPN. On the other hand, the commercial provider of TMA does not deliver all the clinical and histopathological information necessary to establish an adequate association of the markers evaluated with the prognosis of the patients, such as recurrence, survival or follow-up data after diagnosis. Despite this limitations, there are no studies to date evaluating the relationship between CAFs and the tumour expression of PDPN in different forms of HNSCC, although this relationship has already been demonstrated in other types of cancer (32,33). Based on the available evidence and our immunohistochemical findings, a deeper investigation would be useful to understand the role of CAFs and PDPN in EMT.

In conclusion, our investigation showed that a greater presence of CAFs is associated with laryngeal SCC and advanced stages of the disease, possibly because they induce a more aggressive phenotype of the carcinoma, although this would not be related, at least directly, with PDPN expression by the tumour.
